# Corrigenda to Satorra, A., and Bentler, P.M. (2010), “Ensuring Positiveness of the Scaled Difference Chi-Square Test Statistic,” *Psychometrika, 75*, pp. 243–248

**DOI:** 10.1017/psy.2024.12

**Published:** 2025-02-11

**Authors:** Albert Satorra, Peter M. Bentler

**Affiliations:** 1 Universitat Pompeu Fabra, Barcelona; 2 University of California, Los Angeles

On page 245, lines 3 and 4, of the published paper, we find the following text:

“Since 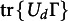 can be expressed as the trace of the product of two positive definite matrices, 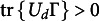 , and thus 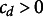 ;”

This text should be replaced with:

“Since 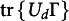 can be expressed as the trace of a positive definite matrix, 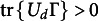 , and thus 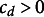 ;”

The uncorrected text claims that 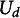 and 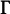 are positive definite matrices, but 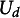 can’t be positive definite, since its rank (difference between the ranks of the derivatives of the two models involved) is much less than its order.

The expression 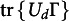 could be written differently so that the conclusion still holds. Namely, write 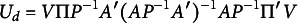 (formula (4) of the paper) as 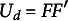 , where 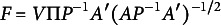 ; then, 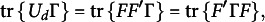 where 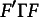 is a positive definite matrix, given that 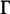 is positive definite in the setup of the paper.

For rewriting the alternative expression of 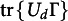 , we used the well-known matrix algebra result that 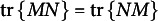 for matrices *M* and *N* of dimensions conformable with the products; in our application, 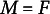 and 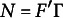 .

